# Two‐Channel Model for Electron Transfer in a Dye‐Catalyst‐Dye Supramolecular Complex for Photocatalytic Water Splitting

**DOI:** 10.1002/cssc.202100846

**Published:** 2021-06-25

**Authors:** Yang Shao, Huub J. M. de Groot, Francesco Buda

**Affiliations:** ^1^ Leiden Institute of Chemistry Leiden University Einsteinweg 55 2300 RA Leiden The Netherlands

**Keywords:** *ab initio* calculations, electron transfer, photocatalysis, photoelectrochemistry, water splitting

## Abstract

To improve the performance of dye‐sensitized photoelectrochemical cell (DS‐PEC) devices for splitting water, the tailoring of the photocatalytic four‐photon water oxidation half‐reaction represents a principle challenge of fundamental significance. In this study, a Ru‐based water oxidation catalyst (WOC) covalently bound to two 2,6‐diethoxy‐1,4,5,8‐diimide‐naphthalene (NDI) dye functionalities provides comparable driving forces and channels for electron transfer. Constrained *ab initio* molecular dynamics simulations are performed to investigate the photocatalytic cycle of this two‐channel model for photocatalytic water splitting. The introduction of a second light‐harvesting dye in the Ru‐based dye‐WOC‐dye supramolecular complex enables two separate parallel electron‐transfer channels, leading to a five‐step catalytic cycle with three intermediates and two doubly oxidized states. The total spin *S*=1 is conserved during the catalytic process and the system with opposite spin on the oxidized NDI proceeds from the Ru=O intermediate to the final Ru‐O_2_ intermediate with a triplet molecular ^3^O_2_ ligand that is eventually released into the environment. The in‐depth insight into the proposed photocatalytic cycle of the two‐channel model provides a strategy for the development of novel high‐efficiency supramolecular complexes for DS‐PEC devices with buildup and conservation of spin multiplicity along the reaction coordinate as a design principle.

## Graphical abstract

**Change the channels**: The inclusion of two channels for the photoinduced electron injection in the photoanode is proposed as a strategy for the development of novel high‐efficiency supramolecular complexes for dye‐sensitized photoelectrochemical cell devices with buildup and conservation of spin multiplicity along the reaction coordinate as a design principle.

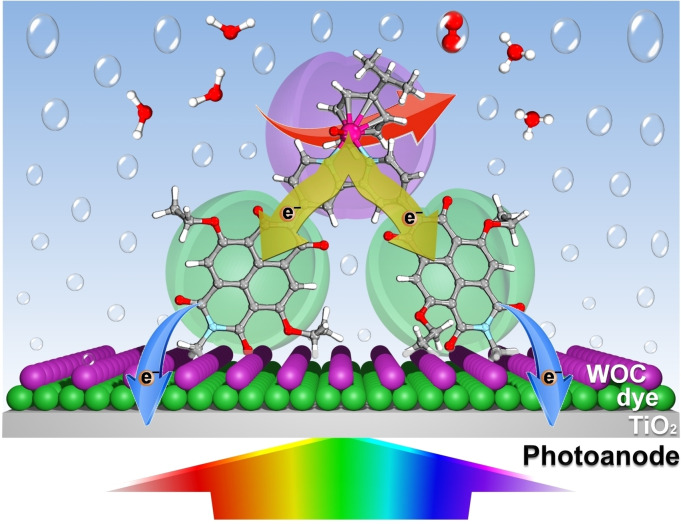

## Introduction

Artificial photosynthesis, inspired by nature, with the goal of conversion of solar energy into chemical energy, has attracted growing interest in the past decades.^[1]^ In particular, dye‐sensitized photoelectrochemical cells (DS‐PECs) that can drive water splitting through the absorption of sunlight, have the potential to produce clean and renewable chemical fuels, e. g. in the form of molecular energy‐rich hydrogen, to meet the future global energy demand in an environmentally sustainable way.^[2]^ Two half reactions are involved when the water splitting process proceeds in two physically separated electrode compartments, the oxygen evolution reaction (OER) for water oxidation, and the hydrogen evolution reaction (HER) for proton reduction.^[3]^ For the catalytic water splitting in DS‐PECs, the high activation free energy barrier for the O−O bond formation process represents a thermodynamic and kinetic bottleneck, and the OER half‐reaction is considered the rate‐determining step.^[4]^

The solar‐driven four‐photon water oxidation half‐reaction occurs at the photoanode, and is always initiated by light absorption at the molecular sensitizers and subsequent electron injection from the dye in the excited state into the electrode.^[5]^ Owing to a proper molecular assembly of the water oxidation catalyst (WOC) and the dye components in a WOC‐dye supramolecular complex, the photooxidation of the dye should be followed by a proton‐coupled electron transfer (PCET)^[6]^ process within the water oxidation catalytic cycle: The electron is used for the regeneration of the dye to its initial state while the proton is being transferred to a different direction, into the environment.^[7]^ Computational studies serve as a powerful technique for guiding the development of efficient dye‐sensitized photoanodes^[8]^ by rate enhancement of photocatalytic water oxidation in DS‐PEC devices and modulation of the mechanism of operation by the solvent environment.^[9]^ A great majority of the computational effort has been devoted to lowering the activation free energy barrier of the catalytic water oxidation step involving the rate‐limiting O−O bond formation process, in which a single channel for the electron transfer (ET) from the WOC to the photooxidized dye was explored.^[9c]^

Since 1970, Kok's classical *S*‐state cycle model of photosynthetic water oxidation involving five oxidation states (*S*_0→4_) has been the paradigm for the understanding of oxygen evolution.^[10]^ By taking into account the role and sequence of deprotonation events as well, an extended *S*‐state cycle has been introduced by Dau et al., in which eight successive steps starting from *I*_0_ lead to *I*_8_ and only then the O_2_ is formed and released.^[11]^ In other words, the *I*‐cycle model involves not only four oxidizing equivalents but also four bases prior to the dioxygen formation. For sequential alternating proton and electron transfer^[7]^ or concerted PCET^[9c]^ according to the Kok or Dau cycle in natural or artificial oxygenic photosynthesis, every individual catalytic PCET step can only proceed after the accomplishment of the previous catalytic step.^[12]^

**Scheme 1:**
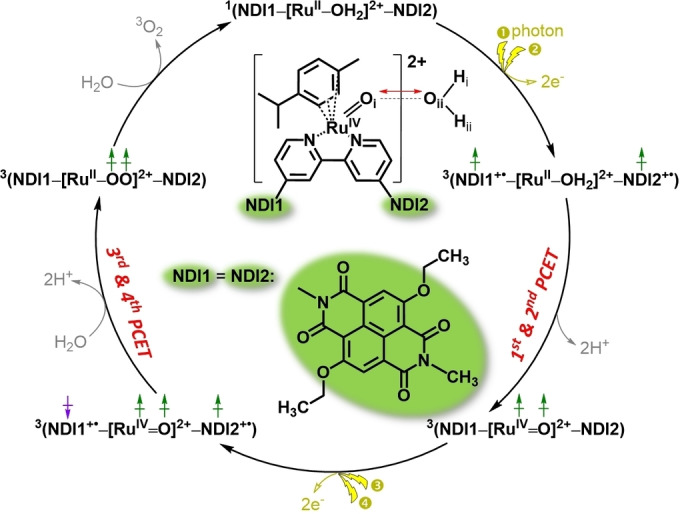
Proposed photocatalytic cycle of the two‐channel model for water splitting by a Ru‐based dye‐WOC‐dye system. The inset shows the structure of the intermediate ^3^(NDI1‐[(cy)Ru^IV^(O)bpy]^2+^‐NDI2) complex (indicated as ^3^(NDI1‐[Ru^IV^=O]^2+^‐NDI2)) together with the attacking water molecule. It is assumed that each light flash induces an electron injection (golden arrows) from NDI1/NDI2 to the semiconductor electrode or to the next stage in a tandem cell, leading to the photooxidation of NDI1/NDI2: NDI1/NDI2→NDI1^+.^/NDI2^+.^. Green (*α* electrons) and purple (*β* electrons) vertical arrows depict the spin of unpaired electrons located on the WOC and on the two NDI dyes. The red double‐sided arrow indicates the reaction coordinate *d*(O_i_←O_ii_) considered in the constrained MD simulations. The superscript on the left indicates the spin multiplicity 2*S*+1 for each intermediate and oxidized state.

In this work we explore how the catalytic cycle could be affected by introducing two excited dye motifs in parallel, and thereby combining two PCET steps without stable intermediates in between. For this aim, an additional dye molecule is introduced in the catalyst‐dye supramolecular complex ^1^([(cy)Ru^II^bpy(H_2_O)]^2+^‐NDI) (cy=*p*‐cymene, bpy=2,2′‐bipyridine, NDI=2,6‐diethoxy‐1,4,5,8‐diimide‐naphthalene) for photocatalytic water splitting, which has been systematically investigated *in silico* recently,[^7^, ^9c^, ^13^] leading to the dye‐WOC‐dye supramolecular complex ^1^(NDI1‐[(cy)Ru^II^bpy(H_2_O)]^2+^‐NDI2) with the total spin *S*=0 (denoted as ^1^(NDI1‐[Ru^II^‐H_2_O]^2+^‐NDI2) in Scheme 1, where NDI1=NDI2=NDI). The goal of this modification is to rearrange the sequence of catalytic intermediates by having first the absorption of two photons, followed by the transfer of two electrons and two protons.

The family of NDI chromophores has shown excellent thermal, chemical and photochemical stability and good photoelectronic properties in various fields, which makes them promising candidates for organic electronics applications, photovoltaic devices, and artificial photosystems.^[14]^ Moreover, the optical properties of the NDI chromophores can be easily tuned over a wide light‐spectrum by adding one or more substituents to the core molecule.^[15]^ Specifically, the NDI dye with diethoxy functional groups considered in this work has been demonstrated to be able to perform fast electron injection in the TiO_2_ semiconductor conduction band on a time scale of picoseconds upon photooxidation.[^5^, ^13a^] The stability of the mononuclear WOC [(cy)Ru^II^bpy(H_2_O)]^2+^ has been investigated using online electrochemical mass spectrometry (OLEMS) and in situ surface enhanced Raman spectroscopy (SERS) in our previous work.^[13b]^ Moreover, the system would be further stabilized once the dye‐WOC‐dye supramolecular complex is anchored on the semiconductor substrate, thus decreasing the possibility of decomposition of the complex during the photocatalytic water splitting reactions.^[16]^ The probability of the absorption of two solar photons and the subsequent injection of two electrons into the semiconductor by the two dyes within the complex on the time scale relevant for the photocatalytic events is quite low in normal light intensity. However, this initial condition can still be realized by using a solar concentrator (antenna) that could considerably enhance the exciton feed‐in rate and thus drive the photochemical processes in parallel. The incorporation of two NDI dye functionalities covalently bound to the bipyridine ligand of the catalytic motif, provides two parallel channels for ET, enabling theoretically concurrent ET events from the WOC to the oxidized dyes NDI1^+.^ and NDI2^+.^. Scheme 1 presents the proposed photocatalytic cycle of the two‐channel model for water splitting by the Ru‐based dye‐WOC‐dye system. An extended photocatalytic cycle considering all possible reaction pathways is shown in Scheme S1 (see the Supporting Information). Given that the spin alignment of unpaired electrons on the WOC and dye has turned out to play a significant role in the PCET reactions in the one‐channel model, only the most favorable pathways with proper spin alignments are explored for the two‐channel model (Scheme 1). Specifically, the first half of the cycle for the two‐channel model is initiated by the co‐photooxidation of two NDI dyes, which leaves one *α* unpaired electron (↑) on each NDI dye with total spin *S*=1. This choice is based on the previous finding for the one‐channel model where the triplet spin configuration was found to be more favorable for the second PCET step. Instead, for the third step involving the O−O bond formation process in the one‐channel model, it is found that the antiparallel spin alignment of the unpaired electrons on the WOC (↑ ↑) and dye (↓) is essential for this reaction. Thus, for the second half of the cycle, the antiparallel spin alignment of unpaired electrons on the two NDI dyes is considered: in this way the total spin *S*=1 is preserved along the whole cycle until the formation of the triplet oxygen (^3^O_2_), which eventually leaves the complex being exchanged by a water molecule and brings the spin multiplicity back to the singlet state (Scheme 1).^[7]^

Herein, we report how the introduction of parallel channels for ET changes the number of involved intermediates and the sequence of reaction events along the photocatalytic cycle in the dye‐WOC‐dye system by using ab initio molecular dynamics (AIMD) simulations, which can provide accurate predictions of the reaction mechanism and activation energy barrier.^[17]^ First, we shortly present the results for the first half of the catalytic cycle. Then, we primarily focus on the second half of the catalytic cycle starting from the second intermediate ^3^(NDI1‐[Ru^IV^=O]^2+^‐NDI2) (Scheme 1, bottom right), since the catalytic step involving the O−O bond formation has long been considered the rate‐limiting step for the photocatalytic water oxidation half‐reaction.

## Results and Discussion

### First half of the catalytic cycle

To verify the feasibility of the entire proposed photocatalytic cycle for the two‐channel model, the first half of the photocatalytic cycle is first investigated. Here we only give the main results (see the Supporting Information, section S2 for details). According to our simulations, the first and second catalytic steps, starting from the initial intermediate ^1^(NDI1‐[Ru^II^‐OH_2_]^2+^‐NDI2) (*S*=0) and ending with the intermediate ^3^(NDI1‐[Ru^IV^=O]^2+^‐NDI2) (*S*=1; see 1^st^ and 2^nd^ PCET in Scheme 1), can proceed with a low activation free energy barrier of around 4 kcal mol^−1^ after the co‐photooxidation of the two NDI dyes (Figure S2). In this process, we assume that one attacking water molecule is approaching H_iii_ while at the same time another attacking water molecule approaches H_iv_ (where H_iii_ and H_iv_ refer to the two hydrogen atoms of the ligated water; Figure S1). The spins are parallel on the NDI in the initial photooxidized state (Figure S1) and the spin on the WOC is built up from *S*=0 to *S*=1 during the two PCET steps.

### Geometry optimization of the dye‐WOC‐dye complex for the second half of the catalytic cycle

The initial geometry of the dye‐WOC‐dye complex ^3^(NDI1‐[Ru^IV^=O]^2+^‐NDI2) was optimized at the DFT level employing the OPBE exchange‐correlation functional^[18]^ and the TZP (triple‐ζ polarized) Slater‐type basis set with the ADF software package^[19]^ (see the Supporting Information S3 for more computational details).^[13a]^ To check if the photooxidized dyes coupled to the Ru‐based WOC exert thermodynamic driving forces for the subsequent catalytic steps, the frontier molecular orbital energy levels together with the singly occupied molecular orbitals (SOMOs) of the doubly‐oxidized complex ^3^(NDI1^+.^‐[Ru^IV^=O]^2+^‐NDI2^+.^) with total spin *S*=1 (Scheme 1) are shown in Figure S3 and the corresponding energy values are listed in Table S2 (see the Supporting Information S4). A closed systems approach simulation^[20]^ with *S*=1 allows to have the same total spin for the initial (^3^[Ru^IV^=O]^2+^) and for the final (^3^[Ru^II^‐O_2_]^2+^) intermediates, thus avoiding the need for intersystem crossing during the reaction: The electronic ground state of the ^3^[Ru^IV^=O]^2+^ WOC is a triplet configuration, whereas the two unpaired electrons on the photooxidized dyes are in an antiparallel arrangement. We also checked the case of the parallel spin configuration for the two unpaired electrons on the dyes, leading to a quintet state ^5^(NDI1^+.^‐[Ru^IV^=O]^2+^‐NDI2^+.^). The result shows that the bond energy difference between the triplet ^3^(NDI1^+.^‐[Ru^IV^=O]^2+^‐NDI2^+.^) and the quintet state ^5^(NDI1^+.^‐[Ru^IV^=O]^2+^‐NDI2^+.^) is negligible (see the Supporting Information S5). This is not surprising considering the relatively large distance between the two dyes, thus an intersystem crossing could easily take place between these two energetically degenerate states.

It is found that the alignment of the energy levels is favorable for the subsequent ET steps involving the O−O bond formation since the SOMOs localized on the NDI dyes (SOMO dye1 and SOMO dye2) with antiparallel spins are lower in energy than the HOMO of the dye‐WOC‐dye complex localized on the WOC (SOMO WOC; Figure S3). The orbital energy difference between the SOMO WOC and the SOMO dyes is Δ*E*_SOMO‐1_≈0.18 and Δ*E*_SOMO‐2_≈0.21 eV, respectively (see Table S2).

### Equilibration of the solvated system and co‐photooxidation of two NDI dyes

An orthorhombic box of dimensions 25.5×22.4×15.4 Å^3^ with periodic boundary conditions containing the dye‐WOC‐dye solute ^3^(NDI1‐[Ru^IV^=O]^2+^‐NDI2) (*S*=1) together with 212 explicit water molecules was used in the AIMD simulations to get accurate predictions of the catalytic reaction and free energy profile. AIMD simulations were carried out with the Car‐Parrinello molecular dynamics (CPMD) program.^[21]^ The electronic structure was determined using GTH pseudopotentials for the ruthenium transition metal^[22]^ and dispersion‐corrected pseudopotentials (DCACP) for the remaining atoms,^[23]^ together with a plane wave cutoff of 70 Ry and the OPBE exchange‐correlation functional (see the Supporting Information for more computational details). The OPBE functional represents a good compromise between the accuracy in the description of transition metal complexes and the computational cost involved in AIMD simulations for such large systems.^[18]^ An initial free AIMD simulation of 0.6 ps at room temperature (300 K) was performed for the solvated system to further equilibrate the solvation environment (see section S3.2).

The system is assumed to be already in its doubly‐oxidized form of dye^+.^‐[WOC]^2+^‐dye^+.^ at the beginning of the constrained AIMD simulation for the second half of the cycle, since the photoinduced electron injection from the selected NDI to a TiO_2_ semiconductor surface can be achieved on a time scale of approximately 1 ps, as has been demonstrated in previous work.[^5^, ^13a^] The co‐photooxidation is mimicked by removing two electrons from the simulation box after the initial equilibration simulation of the dye‐WOC‐dye system leading to a total charge of 4+. A free AIMD simulation for another 0.6 ps at room temperature is performed to further equilibrate the fully oxidized system with total spin *S*=1 corresponding to antiparallel spins on the two NDI dyes. When tracking the spin density along this free AIMD simulation, it is found that two unpaired *α* electrons (↑) localize on the WOC at the Ru^IV^=O group, one unpaired *β* electron (↓) on NDI1, and one unpaired *α* electron (↑) on NDI2 in the doubly oxidized system (see the inset in Figure S5). No ET occurs at this stage, which is an indication of the stability of the initial state of the oxidized complex ^3^(NDI1^+.^‐[Ru^IV^=O]^2+^‐NDI2^+.^) (*S*=1).

### Constrained AIMD simulations and catalytic water oxidation steps

**Figure 1:**
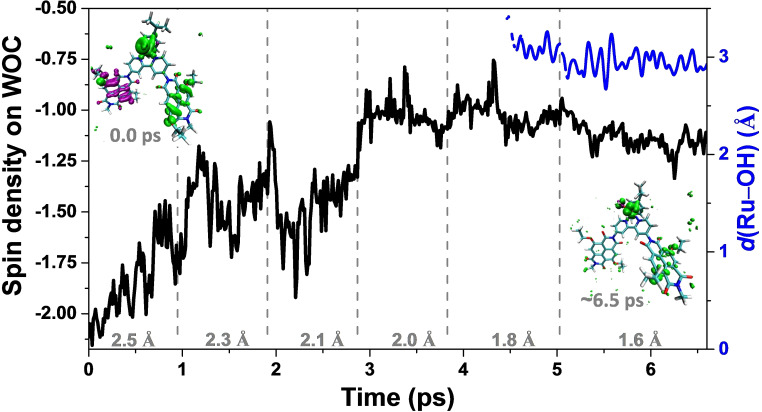
Spin density integrated over the upper half of the simulation box including the WOC (black line) and time evolution of the distance between Ru and the OH *d*(Ru‐OH) (blue line) along the constrained MD trajectories. The OH is defined as an O atom with only one H within a radius of 1.2 Å, illustrating the PT during the MD simulations. Inset left shows the spin density isosurface computed at a snapshot taken at the beginning of the constrained 2.5 Å MD simulation (0.0 ps), clearly indicating two unpaired *α* electrons (↑ in green) localized on the catalyst, one unpaired *β* electron on NDI1 (↓ in purple), and one unpaired α electron on NDI2 (↑ in green). Inset right shows the spin density isosurface computed at the end of the constrained 1.6 Å simulation (ca. 6.5 ps). According to the simulations, one proton of the attacking water is totally released during the constrained 1.6 Å simulation and only oxygen O_ii_ is in the OH form at any time. An integrated spin density of −2 corresponds to two unpaired *α* electrons (↑). The value of the constrained reaction coordinate *d*(O_i_←O_ii_) in the MD simulations is noted in grey. The water molecules are omitted for clarity in both cases and only the initial doubly oxidized state ^3^(NDI1^+.^‐[Ru^IV^=O]^2+^‐NDI2^+.^) (*S*=1) and the transient final state ^3^(NDI1‐[Ru^III^‐OOH]^2+^‐NDI2^+.^) (*S*=1) are shown explicitly. See Scheme 1 for the atomic labeling.

To explore the catalytic water oxidation steps involving bond‐forming and bond‐breaking processes, which are normally considered as rare events on the characteristic AIMD simulation time scale, the constrained MD approach was employed in the simulations to control the reaction coordinate after the re‐equilibration of the photooxidized system.^[24]^ The constrained reaction coordinate in this case is the distance between the oxygen atom O_i_ on the Ru complex and the O_ii_ oxygen of the attacking water indicated by the red double‐sided arrow in Scheme 1 (see the Supporting Information S3 for more computational details). To visualize when and how the electron transfers from the WOC to the oxidized NDI dyes (NDI1 and NDI2 in Scheme 1), the spin density was tracked during the AIMD simulations. The variation of the spin density localized on the WOC (black line) along the constrained MD trajectories is collected in Figure 1. The initial value of −2 for the spin density corresponds to the triplet state with two unpaired electrons on the WOC.

For the two‐channel model starting with the oxidized ^3^(NDI1^+.^‐[Ru^IV^=O]^2+^‐NDI2^+.^) (*S*=1) complex (Figure 1, inset (left)), the ET starts at the reaction coordinate *d*(O_i_←O_ii_)=2.5 Å (Figure 1, black line), whereas in the one‐channel model it was actually observed at the reaction coordinate *d*(O_i_←O_ii_)=2.1 Å.^[7]^ The shortening of *d*(O_i_←O_ii_) from 2.5 Å to 2.0 Å induces the complete ET from the WOC to the oxidized NDI1 with spin density localized on the WOC fluctuating around an average value of −1. After short‐term fluctuations of spin density localized on the WOC, the dye system that is initially in the dye^+.^‐[WOC]^2+^‐dye^+.^ state ends up with one unpaired *α* electron (↑) localized on the WOC and one unpaired *α* electron (↑) on the NDI2 at the end of the constrained 1.8 Å MD simulation. Moreover, the proton transfer (PT) from the attacking water molecule to the solvent is first observed during the constrained 1.8 Å MD simulation when tracking the distance between Ru and the OH *d*(Ru‐OH) along the constrained MD trajectories (Figure 1, blue line): here the OH is defined as an O atom with only one H within a radius of 1.2 Å. Subsequently, the released proton H_i_ diffuses into the solvent bulk via a “chain” of hydrogen‐bonded water molecules following a Grotthuss‐type mechanism[^7^, ^8f^, ^13a^, ^25^] and no back reaction occurs after roughly 5.2 ps along the constrained 1.6 Å MD trajectory (see the Supporting Information S7). It is also noticeable that during the constrained 1.6 Å MD simulation, the integrated spin density gets an average value smaller than −1, which can be attributed to the initial attempts of the fourth ET process from the WOC to the oxidized NDI2 (Figure 1, inset, right).

**Figure 2:**
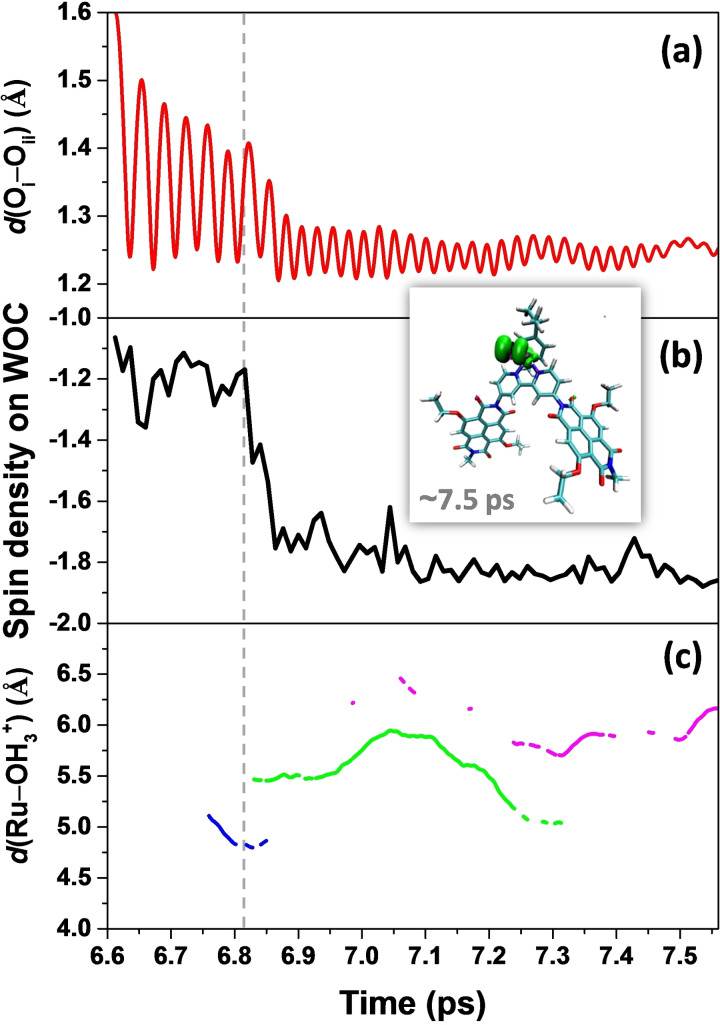
a) Time evolution of the geometrical parameter *d*(O_i_‐O_ii_), b) spin density integrated over upper half of the simulation box including the WOC, and c) distance between Ru and the H_3_O^+^ *d*(Ru‐OH_3_^+^) along the free MD trajectory after releasing the constraint at the end of the constrained 1.6 Å MD simulation. The H_3_O^+^ is defined as an O atom with 3 H within a radius of 1.2 Å, illustrating the second PT during the MD simulation. According to the simulations, only one oxygen is in the H_3_O^+^ form at any time, and the second excess proton associates primarily to three different oxygens (indicated with different colors: blue, green, and magenta) during the simulation. An integrated spin density of −2 corresponds to two unpaired *α* electrons (↑). The inset shows the spin density isosurface computed for a snapshot taken at the end of the free MD simulation, which indicates clearly that the spin density is mostly localized on the ^3^O_2_ ligand and shows the characteristic shape expected for the oxygen molecule, whereas virtually no spin density is observed on the NDIs. The water molecules are omitted for clarity and only the final intermediate NDI1‐[Ru^II^‐OO]^2+^‐NDI2 (*S*=1) is shown explicitly. The time range is consistent with Figure 1. See Scheme 1 for the atomic labeling.

At the end of the constrained 1.6 Å MD simulation, the constraint on the reaction coordinate was released and the system is allowed to evolve freely. The time evolution of the distance between the oxygen atoms O_i_ and O_ii_ *d*(O_i_‐O_ii_), the variation of the total spin density localized on the WOC, and the distance between Ru and H_3_O^+^ (defined as an O atom with three H within a radius of 1.2 Å) along the free MD trajectory after releasing the constraint are collected in Figure 2 for quantitative descriptions of electron and proton dynamics.

Based on these data, the O−O bond distance relaxes within a very short time of about 0.2 ps to an average value of *d*(O_i_‐O_ii_)≈1.35 Å (Figure 2a), which is consistent with the formation of a transient Ru‐OOH state (for comparison, the O−O bond length in molecular hydrogen peroxide is 1.47 Å). After 0.2 ps (at ca. 6.8 ps; Figure 2, dashed vertical line) a fast ET process from the WOC to the oxidized NDI2 takes place (Figure 2b). This ET process is strongly coupled to the fourth PT from the hydroperoxo ligand to the solvent bulk (Figure 2c). Notice that two protons (H_i_ and H_ii_) diffuse independently from each other into the solvent at this stage and we only focus on the second released proton H_ii_ in Figure 2c. The distance between the oxygen atoms O_i_ and O_ii_ equilibrates quickly to an average value *d*(O_i_‐O_ii_) of about 1.25 Å. Although we have a higher proton density compared to the one‐channel model, we observe that the fourth PCET catalytic water oxidation step proceeds spontaneously following the formation of the O−O bond and the system reaches the final intermediate ^3^(NDI1‐[Ru^II^‐OO]^2+^‐NDI2) [*S*=1; Scheme 1 and Eq. (1), where H_2_O_sol_ and H^+^_sol_ represent the solvated attacking water molecule and solvated proton respectively].

(1) $${}^{3}\left(\mathrm{NDI}1{}^{+}{}^{•}\text{-}\left[\mathrm{Ru}{}^{\mathrm{IV}}=O\right]{}^{2+}\text{-}\mathrm{NDI}2{}^{+}{}^{•}\right)+H{}_{2}O{}_{\mathrm{sol}}↔{}^{3}\left(\mathrm{NDI}1\text{-}\left[\mathrm{Ru}{}^{\mathrm{II}}\text{-}\mathrm{OO}\right]{}^{2+}\text{-}\mathrm{NDI}2\right)+2H{}^{+}{}_{\mathrm{sol}}$$

**Scheme 2:**
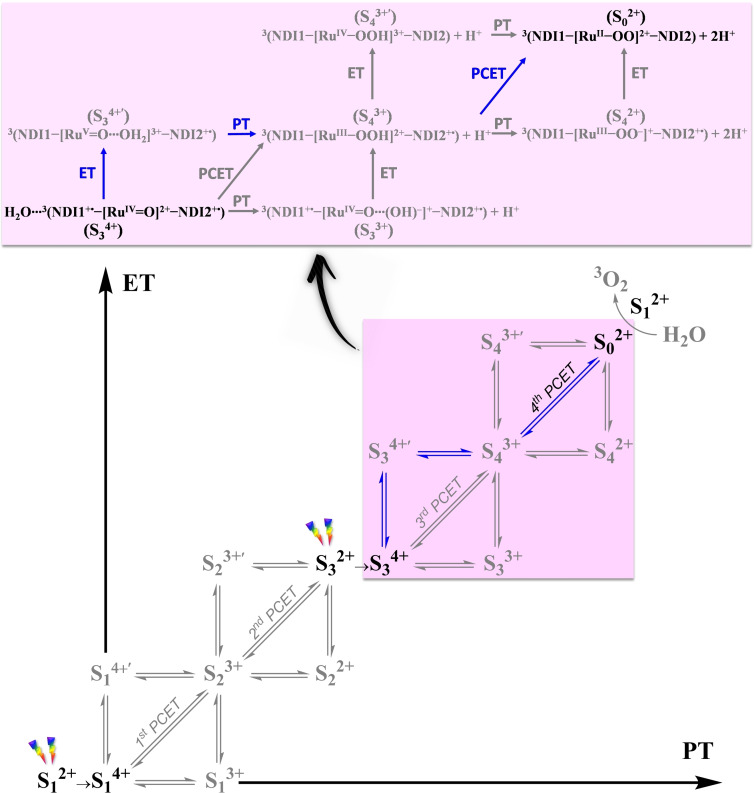
Four PCET steps between the catalytic intermediates from S_1_^2+^ to S_0_^2+^ for the supramolecular dye‐WOC‐dye complex. It is assumed that two light flashes induce the co‐photooxidation of the two NDI dyes in the two‐channel model (S_i_^2+^→S_i_^4+^, i=0–4: NDI1→NDI1^+.^ and NDI2→NDI2^+.^). The vertical and horizontal double arrows correspond to the pathways of a sequential PCET mechanism, either ET from the WOC to the oxidized dye first (S_i_^4+^→S_i_^4+^′ and S_i_^3+^→S_i_^3+^′) or PT to the solvent first (S_i_^4+^→S_i_^3+^ and S_i_^3+^→S_i_^2+^). The diagonal double arrow denotes the concerted PCET mechanism. The favorable pathway of the second half of the catalytic cycle in the two‐channel model is indicated in blue. The stable intermediates investigated in the present study are shown in black. The ligand exchange S_0_^2+^+H_2_O→S_1_^2+^+^3^O_2_ is also indicated in grey. H^+^ represents the proton transferred to the solvent. The catalytic steps from S_3_^4+^ to S_0_^2+^, which are the main focus of this work, are specifically described in the top panel.

In this final complex with an average *d*(O_i_‐O_ii_)≈1.25 Å two unpaired *α* electrons (↑) are localized on the dioxygen ligand (Figure 2, inset). This compares well with the bond length 1.21 Å in molecular ^3^O_2_ in its triplet state and indicates the formation of the oxygen‐oxygen bond in the triplet state featuring a bond order of 2 as in molecular oxygen. The ^3^O_2_ ligand can then be exchanged by a water molecule and the complex is ready for the next catalytic cycle. All these results indicate that the third and fourth catalytic steps proceed in a sequential way without a stable intermediate between these two steps (see S_3_^4+^→S_0_^2+^ in Scheme 2).

**Scheme 3:**
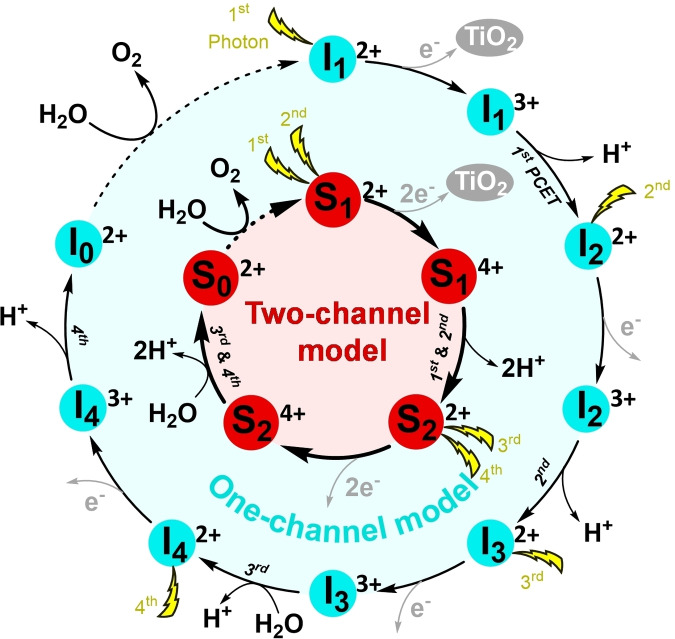
Basic photocatalytic cycle of the one‐channel and two‐channel models for water splitting by WOC‐dye and dye‐WOC‐dye systems, respectively. The photocatalytic cycle of the one‐channel model by a Ru‐based WOC‐dye complex includes nine stable states (outer circle), in which eight successive electron transfer and proton transfer steps starting from I_1_^2+^ lead to I_0_^2+^ and only then the O_2_ is formed and released.[^7^, ^13a^] The photocatalytic cycle of the two‐channel model by a Ru‐based dye‐WOC‐dye complex includes five stable states from S_1_^2+^ to S_0_^2+^ (inner circle). The superscript on the right indicates the total charge of the supramolecular complex.

This result is at variance with the case of the one‐channel model where the complex with a hydroperoxo ligand was found to be a stable intermediate (see I_4_^2+^ and I_4_^3+^ in Scheme 3), whereas here it is only a transient Ru−OOH state developing into the final intermediate (Scheme 2).^[7]^

### Free energy profile and reaction rate evaluation

**Figure 3:**
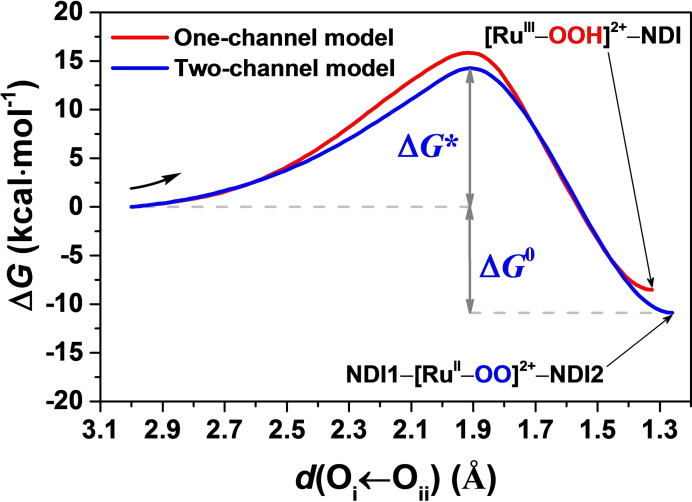
Free energy profile along the reaction coordinate *d*(O_i_←O_ii_) computed by thermodynamic integration. The final intermediates corresponding with the MD simulations for the one‐channel and two‐channel models are both indicated. The free energy profile obtained for the one‐channel model from a previous study is also presented for comparison (see Ref. [7]).

Having established that the second half of the catalytic water oxidation cycle starting from the doubly photooxidized supramolecular complex ^3^(NDI1^+^‐[Ru^IV^=O]^2+^‐NDI2^+^) (*S*=1) proceeds combining two sequential steps without stable intermediates in between, it is relevant to evaluate how difficult it is to activate this reaction in such a two‐channel model. In recent years, metadynamics simulations have been increasingly used as an alternative enhanced sampling method in similar computational works on O−O bond formation, which allows to sample the entire free energy landscape.^[26]^ Particularly, a second collective variable has been included to keep track of the proton transfer in addition to the O−O distance in very recent publications by Luber et al.[^26c^, ^26d^] In our current work instead, the reaction coordinate *d*(O_i_←O_ii_) is constrained to a series of fixed values to estimate the free energy profile along this reaction pathway using the Blue Moon ensemble approach and thermodynamic integration.[^24^, ^27^] The time‐averaged mean forces associated with the applied constraints and the interpolation of the time‐averaged mean forces used for this analysis, and the corresponding free energy profile of the two‐channel model as a function of the reaction coordinate *d*(O_i_←O_ii_) are given in Figure S7 and Figure 3, respectively (see the Supporting Information S3.3 for more computational details). Table 1 summarizes the thermodynamic parameters for the O−O bond formation process extracted from these results.

**Table 1:** The calculated activation energy barrier (ΔG* in kcal mol^−1^), reaction driving force (Δ*G* ^0^ in kcal mol^−1^), and the reaction rate (*k* in s^−1^) corresponding to the one‐ and two‐channel models. The results for the one‐channel model are taken from Ref. [7].

Model	ΔG*	Δ*G* ^0^	*k*
One‐channel^[7]^	15.9	−8.5	15.7
Two‐channel	14.3	−10.9	230.4

The calculated activation free energy barrier (ΔG*) for the two‐channel model is 14.3 kcal mol^−1^ (ca. 0.62 eV), which is slightly lower than the 15.9 kcal mol^−1^ (ca. 0.69 eV) computed with the same approach for the one‐channel model^[7]^ (see Table 1). However, this conclusion might be affected by the statistical error in the time‐averaged mean forces. If we use this barrier for the estimation of the reaction rate according to transition state theory^[28]^ (see the Supporting Information S3.4 for computational details), the predicted reaction rate of the two‐channel model is *k*=230.4 s^−1^, which is faster than that obtained for the one‐channel model (*k*=15.7 s^−1^). Introducing one proton acceptor group near the active site could further lower the activation free energy barrier and thus accelerate the O−O bond formation.^[9c]^ Alternative WOCs in which the ligand has been functionalized with proton acceptor groups, e. g. carboxylate moieties, can facilitate the water splitting reaction.^[8e]^ One should keep in mind that the two‐channel model ends up with the final intermediate ^3^(NDI1‐[Ru^II^‐OO]^2+^‐NDI2) (*S*=1; Scheme 1 and Figure 3) rather than an intermediate with a hydroperoxo ligand as in the one‐channel model (^2^([Ru^III^‐OOH]^2+^‐NDI) *S*=1/2)^[7]^ as a result of the introduction of the second electron‐transfer channel.

In addition, the larger thermodynamic driving force Δ*G* ^0^=−10.9 kcal mol^−1^ (ca. 0.47 eV) obtained for the two‐channel model can be reasonably attributed to the accomplishment of the barrier‐less fourth catalytic water splitting PCET step under the condition that the second dye NDI2 is photooxidized. This result suggests a relatively stable final intermediate ^3^(NDI1‐[Ru^II^‐OO]^2+^‐NDI2) (*S*=1; Scheme 1) that is lower in energy than an alternative in‐between intermediate ^3^(NDI1‐[Ru^III^‐OOH]^2+^‐NDI2^+.^) (*S*=1; Scheme S1). Finally, the cycle is completed by replacing the dissociating triplet molecular oxygen with a water molecule, leading to the initial singlet ^1^(NDI1‐[Ru^II^‐OH_2_]^2+^‐NDI2) state. Hence, the Ru metal ion operates as a spin shuttle during catalysis. In the first part of the cycle, it selects the matching spin from the NDI to build up spin multiplicity, and in the second part it preserves the spin multiplicity and passes on a triplet to the oxygen.

## Conclusion

In conclusion, the introduction of the second NDI dye in the dye‐WOC‐dye complex for photocatalytic water splitting provides an extra channel for ET, which enables the sequential event of ET from the WOC to the two separate NDI dyes. The dynamical description of the proposed photocatalytic cycle of the two‐channel model obtained with adiabatic AIMD simulations and explicit solvation demonstrates that the third and fourth catalytic steps can proceed one after the other without stable intermediates in between. Although the estimated activation free energy barrier of the combined third and fourth catalytic steps for the two‐channel model is similar to that of the one‐channel model, the introduction of the second ET channel removes one intermediate in the cycle: the system can now proceed without changing the total spin of the supramolecular complex, from the Ru=O intermediate to the final intermediate with a triplet molecular ^3^O_2_ product. Overall, this study suggests that having the WOC coordinated to more than one dye at the photoanode of a DS‐PEC device can have beneficial effects in the rate and efficiency of the photocatalytic cycle: this is achieved by having the co‐photooxidation of the two dyes and an antiparallel spin alignment of the unpaired electrons on the dyes.

## Conflict of interest

The authors declare no conflict of interest.

## Supporting information

As a service to our authors and readers, this journal provides supporting information supplied by the authors. Such materials are peer reviewed and may be re‐organized for online delivery, but are not copy‐edited or typeset. Technical support issues arising from supporting information (other than missing files) should be addressed to the authors.

Supporting Information

## References

[1a] M. Grätzel, Nature 2001, 414, 338–344; 11713540 10.1038/35104607

[1b] S. Berardi, S. Drouet, L. Francas, C. Gimbert-Surinach, M. Guttentag, C. Richmond, T. Stoll, A. Llobet, Chem. Soc. Rev. 2014, 43, 7501–7519. 24473472 10.1039/c3cs60405e

[2a] S. Zhang, H. Ye, J. Hua, H. Tian, EnergyChem 2019, 1, 100015;

[2b] S. Yun, N. Vlachopoulos, A. Qurashi, S. Ahmad, A. Hagfeldt, Chem. Soc. Rev. 2019, 48, 3705–3722; 31120048 10.1039/c8cs00987b

[2c] Z. N. Zahran, Y. Tsubonouchi, E. A. Mohamed, M. Yagi, ChemSusChem 2019, 12, 1775–1793; 30793506 10.1002/cssc.201802795

[2d] P. Xu, N. S. McCool, T. E. Mallouk, Nano Today 2017, 14, 42–58;

[2e] M. K. Brennaman, R. J. Dillon, L. Alibabaei, M. K. Gish, C. J. Dares, D. L. Ashford, R. L. House, G. J. Meyer, J. M. Papanikolas, T. J. Meyer, J. Am. Chem. Soc. 2016, 138, 13085–13102. 27654634 10.1021/jacs.6b06466

[3a] Z. Yu, F. Li, L. Sun, Energy Environ. Sci. 2015, 8, 760–775;

[3b] X. Ding, L. Zhang, Y. Wang, A. Liu, Y. Gao, Coord. Chem. Rev. 2018, 357, 130–143.

[4a] M. E. G. Lyons, R. L. Doyle, M. P. Browne, I. J. Godwin, A. A. S. Rovetta, Curr. Opin. Electrochem. 2017, 1, 40–45;

[4b] P. Xu, T. Huang, J. Huang, Y. Yan, T. E. Mallouk, Proc. Natl. Acad. Sci. USA 2018, 115, 6946–6951. 29915092 10.1073/pnas.1804728115 PMC6142270

[5] J. P. Menzel, A. Papadopoulos, J. Belić, H. J. M. de Groot, L. Visscher, F. Buda, J. Phys. Chem. C 2020, 124, 27965–27976.

[6a] S. Hammes-Schiffer, Chem. Rev. 2010, 110, 6937–6938; 21141827 10.1021/cr100367q

[6b] C. J. Gagliardi, A. K. Vannucci, J. J. Concepcion, Z. Chen, T. J. Meyer, Energy Environ. Sci. 2012, 5, 7704–7717;

[6c] S. Hammes-Schiffer, J. Am. Chem. Soc. 2015, 137, 8860–8871; 26110700 10.1021/jacs.5b04087 PMC4601483

[6d] S. Horvath, L. E. Fernandez, A. V. Soudackov, S. Hammes-Schiffer, Proc. Natl. Acad. Sci. USA 2012, 109, 15663–15668; 22529352 10.1073/pnas.1118333109 PMC3465444

[6e] B. H. Solis, S. Hammes-Schiffer, Inorg. Chem. 2014, 53, 6427–6443. 24731018 10.1021/ic5002896

[7] Y. Shao, J. M. de Ruiter, H. J. M. de Groot, F. Buda, J. Phys. Chem. C 2019, 123, 21403–21414.

[8a] J. R. Swierk, T. E. Mallouk, Chem. Soc. Rev. 2013, 42, 2357–2387; 23081721 10.1039/c2cs35246j

[8b] D. Wang, M. S. Eberhart, M. V. Sheridan, K. Hu, B. D. Sherman, A. Nayak, Y. Wang, S. L. Marquard, C. J. Dares, T. J. Meyer, Proc. Natl. Acad. Sci. USA 2018, 115, 8523–8528; 30082396 10.1073/pnas.1802903115 PMC6112685

[8c] S. Maji, L. Vigara, F. Cottone, F. Bozoglian, J. Benet-Buchholz, A. Llobet, Angew. Chem. Int. Ed. 2012, 51, 5967–5970; 10.1002/anie.201201356 22549816

[8d] P. Garrido-Barros, I. Funes-Ardoiz, S. Drouet, J. Benet-Buchholz, F. Maseras, A. Llobet, J. Am. Chem. Soc. 2015, 137, 6758–6761; 25984748 10.1021/jacs.5b03977

[8e] R. Matheu, M. Z. Ertem, J. Benet-Buchholz, E. Coronado, V. S. Batista, X. Sala, A. Llobet, J. Am. Chem. Soc. 2015, 137, 10786–10795; 26226390 10.1021/jacs.5b06541

[8f] Y. Shao, H. J. M. de Groot, F. Buda, ChemSusChem 2021, 14, 479–486. 32871047 10.1002/cssc.202001863 PMC7821158

[9a] N. Song, J. J. Concepcion, R. A. Binstead, J. A. Rudd, A. K. Vannucci, C. J. Dares, M. K. Coggins, T. J. Meyer, Proc. Natl. Acad. Sci. USA 2015, 112, 4935–4940; 25848035 10.1073/pnas.1500245112 PMC4413278

[9b] Z. Chen, J. J. Concepcion, X. Hu, W. Yang, P. G. Hoertz, T. J. Meyer, Proc. Natl. Acad. Sci. USA 2010, 107, 7225–7229; 20360565 10.1073/pnas.1001132107 PMC2867729

[9c] Y. Shao, H. J. M. de Groot, F. Buda, J. Phys. Chem. Lett. 2019, 7690–7697. 31763842 10.1021/acs.jpclett.9b02914 PMC6926955

[10] B. Kok, B. Forbush, M. McGloin, Photochem. Photobiol. 1970, 11, 457–475. 5456273 10.1111/j.1751-1097.1970.tb06017.x

[11] H. Dau, M. Haumann, Biochim. Biophys. Acta Bioenerg. 2007, 1767, 472–483. 10.1016/j.bbabio.2007.02.022 17442260

[12] M. Haumann, P. Liebisch, C. Müller, M. Barra, M. Grabolle, H. Dau, Science 2005, 310, 1019–1021. 16284178 10.1126/science.1117551

[13a] A. Monti, J. M. de Ruiter, H. J. M. de Groot, F. Buda, J. Phys. Chem. C 2016, 120, 23074–23082;

[13b] J. M. de Ruiter, R. L. Purchase, A. Monti, C. J. M. van der Ham, M. P. Gullo, K. S. Joya, M. D'Angelantonio, A. Barbieri, D. G. H. Hetterscheid, H. J. M. de Groot, F. Buda, ACS Catal. 2016, 6, 7340–7349.

[14a] M. Al Kobaisi, S. V. Bhosale, K. Latham, A. M. Raynor, S. V. Bhosale, Chem. Rev. 2016, 116, 11685–11796; 27564253 10.1021/acs.chemrev.6b00160

[14b] A. Diac, M. Matache, I. Grosu, N. D. Hădade, Adv. Synth. Catal. 2018, 360, 817–845.

[15] J. Belić, B. van Beek, J. P. Menzel, F. Buda, L. Visscher, J. Phys. Chem. A 2020, 124, 6380–6388. 32649188 10.1021/acs.jpca.0c04506 PMC7416309

[16] M. de Respinis, K. S. Joya, H. J. M. De Groot, F. D'Souza, W. A. Smith, R. van de Krol, B. Dam, J. Phys. Chem. C 2015, 119, 7275–7281.

[17] D. Marx, J. Hutter, *Ab Initio Molecular Dynamics: Basic Theory and Advanced Methods*, Cambridge University Press, Cambridge, **2009**.

[18a] M. Swart, A. W. Ehlers, K. Lammertsma, Mol. Phys. 2004, 102, 2467–2474;

[18b] A. T. P. Carvalho, M. Swart, J. Chem. Inf. Model. 2014, 54, 613–620; 24460186 10.1021/ci400718m

[18c] A. R. Groenhof, A. W. Ehlers, K. Lammertsma, J. Am. Chem. Soc. 2007, 129, 6204–6209; 17441718 10.1021/ja0685654

[18d] J. Conradie, A. Ghosh, J. Chem. Theory Comput. 2007, 3, 689–702; 26627386 10.1021/ct600337j

[18e] J. L. Vallés-Pardo, M. C. Guijt, M. Iannuzzi, K. S. Joya, H. J. M. de Groot, F. Buda, ChemPhysChem 2012, 13, 140–146. 22223632 10.1002/cphc.201100546

[19a] G. te Velde, F. M. Bickelhaupt, E. J. Baerends, C. Fonseca Guerra, S. J. A. van Gisbergen, J. G. Snijders, T. Ziegler, J. Comput. Chem. 2001, 22, 931–967;

[19b] ADF2017, SCM, Theoretical Chemistry, Vrije Universiteit, Amsterdam, The Netherlands, http://www.scm.com.

[20] J. M. de Ruiter, F. Buda, Phys. Chem. Chem. Phys. 2017, 19, 4208–4215. 28054677 10.1039/c6cp07454e

[21] CPMD, http://www.cpmd.org, Copyright IBM Corp., 1990–2019; Copyright MPI für Festkörperforschung Stuttgart, 1997–2001.

[22] C. Hartwigsen, S. Goedecker, J. Hutter, Phys. Rev. B 1998, 58, 3641–3662.

[23] I. C. Lin, M. D. Coutinho-Neto, C. Felsenheimer, O. A. von Lilienfeld, I. Tavernelli, U. Rothlisberger, Phys. Rev. B 2007, 75, 205131.

[24] G. Ciccotti, M. Ferrario, Mol. Simul. 2004, 30, 787–793.

[25a] N. Agmon, Chem. Phys. Lett. 1995, 244, 456–462;

[25b] C. J. T. de Grotthuss, Biochim. Biophys. Acta Bioenerg. 2006, 1757, 871–875. 10.1016/j.bbabio.2006.07.004 16962993

[26a] J. L. Vallés-Pardo, M. C. Guijt, M. Iannuzzi, K. S. Joya, H. J. M. de Groot, F. Buda, ChemPhysChem 2012, 13, 140–146; 22223632 10.1002/cphc.201100546

[26b] S. Piccinin, A. Sartorel, G. Aquilanti, A. Goldoni, M. Bonchio, S. Fabris, Proc. Natl. Acad. Sci. USA 2013, 110, 4917–4922; 23479603 10.1073/pnas.1213486110 PMC3612603

[26c] M. Schilling, R. A. Cunha, S. Luber, J. Chem. Theory Comput. 2020, 16, 2436–2449; 32207933 10.1021/acs.jctc.9b01207

[26d] M. Schilling, R. A. Cunha, S. Luber, ACS Catal. 2020, 10, 7657–7667.

[27a] B. Ensing, E. J. Meijer, P. E. Blöchl, E. J. Baerends, J. Phys. Chem. A 2001, 105, 3300–3310;

[27b] F. Costanzo, R. G. Della Valle, J. Phys. Chem. B 2008, 112, 12783–12789. 18793011 10.1021/jp801702v

[28a] H. Eyring, J. Chem. Phys. 1935, 3, 107–115;

[28b] K. J. Laidler, M. C. King, J. Phys. Chem. 1983, 87, 2657–2664;

[28c] E. Pollak, P. Talkner, Chaos 2005, 15, 026116. 10.1063/1.1858782 16035918

