# Supplementary material for: Two‐Channel Model for Electron Transfer in a Dye‐Catalyst‐Dye Supramolecular Complex for Photocatalytic Water Splitting
Source: ChemSusChem. 2021 Jun 25;14(15):3155–62. doi: 10.1002/cssc.202100846 (PMC8453919; doi:10.1002/cssc.202100846)
Supplement: Supplementary file 1 — Supporting Information [file CSSC-14-3155-s001.pdf]

ChemSusChem

Supporting Information

Two-Channel Model for Electron Transfer in a Dye-Catalyst-Dye Supramolecular Complex for Photocatalytic Water Splitting

Yang Shao,* Huub J. M. de Groot, and Francesco Buda*© 2021 The Authors. ChemSusChem published by Wiley-VCH GmbH. This is an open access article under the terms of the Creative Commons Attribution License, which permits use, distribution and reproduction in any medium, provided the original work is properly cited.

Table of Contents

S1. Expanded reaction pathways S2

S2. First half of the catalytic cycle S3

S3. COMPUTATIONAL DETAILS S8

S3.1 Geometry Optimization at DFT Level S8

S3.2 Simulation Box S8

S3.3 Free Energy Profile S8

S3.4 Reaction Rate S9

S4. Molecular orbital and electronic structure of $^{3}(NDI1^{+\bullet}–[Ru^{IV}=O]^{2+}–NDI2^{+\bullet})$ S10

S5. DFT calculation of $^{5}(NDI1^{+\bullet}–[Ru^{IV}=O]^{2+}–NDI2^{+\bullet})$ S12

S6. Spin density after photooxidation of two NDI dyes S13

S7. Proton transfer (PT) step during the constrained 1.8 Å simulation S13

S8. Time-averaged constraint forces S14

References S15

S1. Expanded reaction pathways

Scheme S1. Expanded Reaction Pathways of the Two-Channel Model by a Ru-based dye–WOC–dye System[a]

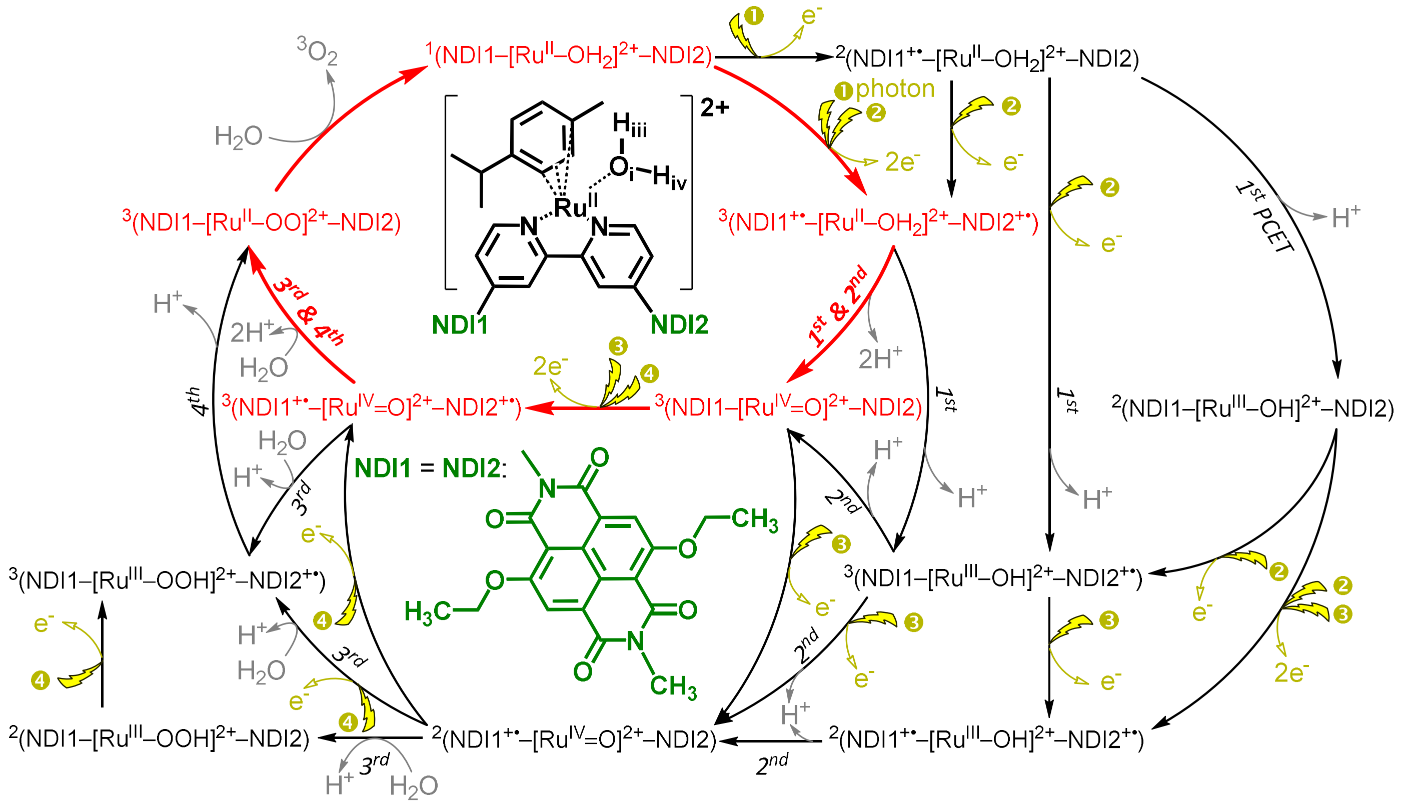

[a]Schematic Structure of the starting intermediate ^{1}([(cy)Ru^{II}bpy(H$_2$O)]$^{2+}$–(NDI)$_2$) complex (indicated shortly as ^{1}(NDI1–[Ru^{II}–OH$_2$]$^{2+}$–NDI2)) on the top of the scheme, as explicitly shown in the inset. It is assumed that each light flash induces an electron injection (golden arrows) from the NDI1/NDI2 to the semiconductor electrode or to the next stage in a tandem cell, leading to the photooxidation of NDI1/NDI2: NDI1/NDI2 → NDI1$^{+•}$/NDI2$^{+•}$. All the possible pathways for the dye−WOC−dye complex after introducing an extra NDI dye are considered in this scheme. In the current work we only focus on the case involving the cophotooxidation of two NDI dyes and the stable intermediates involved as shown in Scheme 1 are indicated in red. The transient states indicated in black are not investigated in this work.

S2. First half of the catalytic cycle

The initial geometry of the dye−WOC−dye complex ^{1}(NDI1−[Ru^{II}−OH$_2$]$^{2+}$−NDI2) (S = 0) was optimized at the DFT level employing the OPBE exchange-correlation functional[1] and the TZP (triple-ζ polarized) Slater-type basis set[2] with the ADF software package[3]. In the geometry optimization, the continuous solvation model (COSMO[4]) for water was used.

To obtain a realistic description of the catalytic reaction step, the solvent was explicitly introduced in the simulations. An orthorhombic box of dimensions 26.5 × 20.1 × 16.3 Å^3 with periodic boundary conditions containing the dye−WOC−dye solute ^{1}(NDI1−[Ru^{II}−OH$_2$]$^{2+}$−NDI2) (S = 0) together with 222 explicit water molecules was used in the AIMD simulations to get accurate predictions of the catalytic reaction, which was carried out with the CPMD program.[5]

Two reaction coordinates were considered in our constrained MD simulations, corresponding to the distances between H_{iii}/H_{iv} and the oxygen atom of its neighboring water molecule (see the atomic labeling in Scheme S1 and Figure S1). The reaction coordinates $d(H_{iii}\leftarrow O)$ as well as $d(H_{iv}\leftarrow O)$ are constrained to a series of fixed values x in range of 1.4 − 1.05 Å simultaneously after the initial equilibrium simulation and subsequent photooxidation of two NDI dyes. In this way, we assume that one attacking water molecule approaches to H_{iii} and another attacking water molecule to H_{iv} at the same time. The photooxidation of the NDI dyes (NDI1 and NDI2) was mimicked by removing two electrons from the simulation box after the initial equilibration simulation of the dye−WOC−dye system, after which a free MD (FMD) simulation of around 1.1 ps at room temperature was performed to equilibrate the oxidized state ^{3}(NDI1$^{+\bullet}$−[Ru^{II}−OH$_2$]$^{2+}$−NDI2$^{+\bullet}$) with the total spin S = 1. According to the results of our simulations, one unpaired α electron (↿) is observed to localize on NDI1 and one unpaired α electron (↿) on NDI2 in the system (see inset (i) in Figure S1). This spin density confirms that

we have obtained the ground state of the oxidized complex ^{3}(NDI1$^{+\bullet}$–[Ru^{II}–OH$_2$]$^{2+}$– NDI2$^{+\bullet}$) (S = 1).

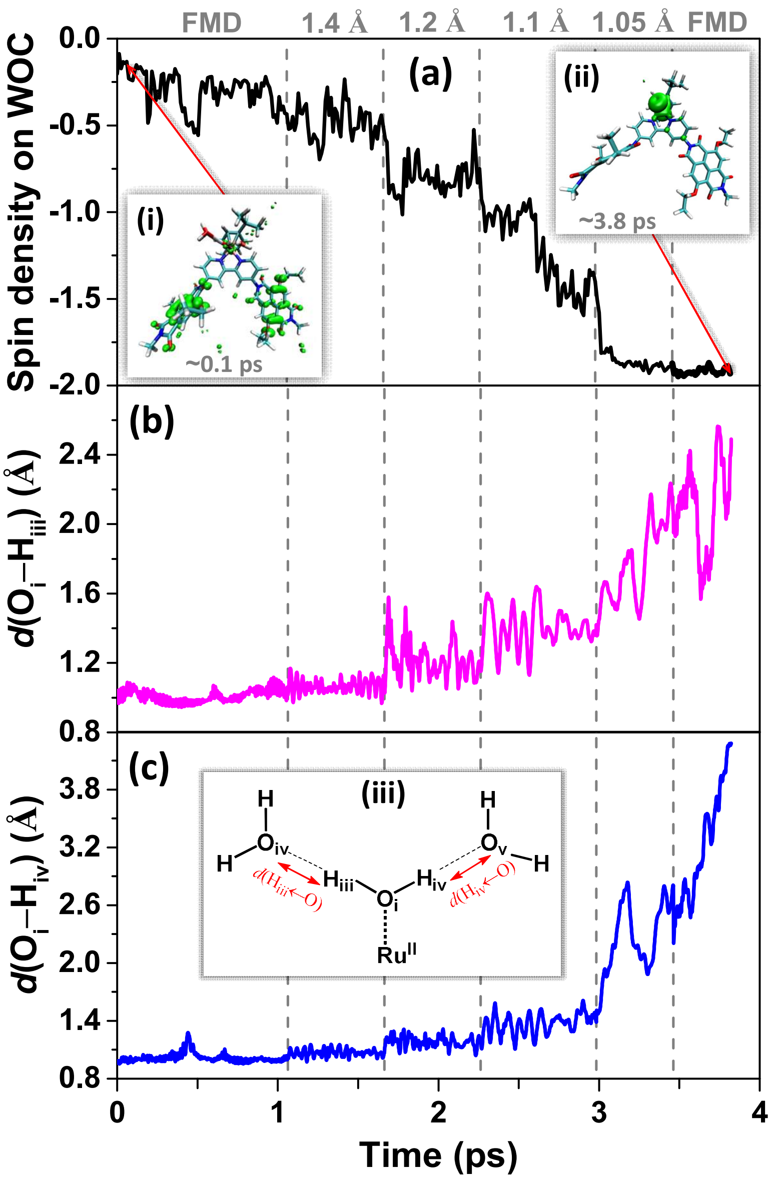

Figure S1. (a) Spin density integrated over the part of the simulation box including the WOC, time evolution of the geometrical parameters (b) d(O$_i$–H$_{iii}$) and (c) d(O$_i$–H$_{iv}$) along the free/constrained MD trajectories after the photooxidation of two NDI dyes. The inset (i) shows the spin density isosurface computed for a snapshot taken at ~0.1 ps, clearly indicating the intermediate ^{3}(NDI1$^{+\bullet}$–[Ru^{II}–OH$_2$]$^{2+}$–NDI2$^{+\bullet}$) with one unpaired α electron (↿ in green) localized on each NDI dye. Inset (ii) shows the spin density isosurface computed for a snapshot taken at the end of the free MD (FMD) simulation, ~3.8 ps, clearly indicating the final intermediate ^{3}(NDI1–[Ru^{IV}=O]$^{2+}$–NDI2) with two unpaired α electrons (↿ in green) localized on the WOC. Inset (iii) shows the schematic structure of the first two water molecules along the hydrogen-bonding network coordinated to the ligand water molecule. For clarity, only the supramolecular complex and the attacking water molecules are shown explicitly. An integrated spin density value of −2 corresponds to two unpaired α electrons (↿). The value of the constrained reaction coordinate d(H$_{iii}$←O)/d(H$_{iv}$←O) in the MD simulations is noted in grey.

The shortening of $d(H_{iii}{\leftarrow}O)$ and $d(H_{iv}{\leftarrow}O)$ from 1.4 Å to 1.1 Å induces the electron transfer from the WOC to the oxidized NDI dyes (see Figure S1a) The $O_i{-}H_{iii}$ and $O_i{-}H_{iv}$ bonds (see inset (iii) in Figure S1 for the atomic labeling) finally break when we further shorten the $H_{iii}{\cdots}O_{iv}$ and $H_{iv}{\cdots}O_v$ distances to 1.05 Å (see Figure S1b and S1c), which occurs almost at the same time as the accomplishment of the electron transfer (see Figure S1a). No back-transfer of either an electron or a proton is observed after the release of the constraints from the system following the constrained 1.05 Å simulation. This confirms the stability of the final product $^3(NDI1{-}[Ru^{IV}{=}O]^{2+}{-}NDI2)$ ($S = 1$) with two unpaired α electrons localized on the WOC after the first and second catalytic PCET steps (see inset (ii) in Figure S1). All these results indicate that the first and second catalytic steps are able to proceed and complete at the same stage after the photooxidation of two NDI dyes.

Based on all the constrained MD simulations performed, the free energy profile along the reaction coordinates $d(H_{iii}{\leftarrow}O)$ and $d(H_{iv}{\leftarrow}O)$ of the oxidized complex $^3(NDI1^{+\bullet}{-}[Ru^{II}{-}OH_2]^{2+}{-}NDI2^{+\bullet})$ ($S = 1$) can be computed using the Bluemoon ensemble approach.[6] The time-averaged forces associated with the applied constraints $d(H_{iii}{\leftarrow}O)$ and $d(H_{iv}{\leftarrow}O)$, the interpolation of the time-averaged mean forces used for this analysis, and the corresponding free energy profiles obtained via thermodynamic integration of the oxidized complex $^3(NDI1^{+\bullet}{-}[Ru^{II}{-}OH_2]^{2+}{-}NDI2^{+\bullet})$ ($S = 1$) are presented in Figure S2. Table S1 summarizes the key thermodynamic parameters extracted from the free energy profiles corresponding to the reaction coordinates $d(H_{iii}{\leftarrow}O)$ and $d(H_{iv}{\leftarrow}O)$ for the first half of the catalytic water oxidation cycle.

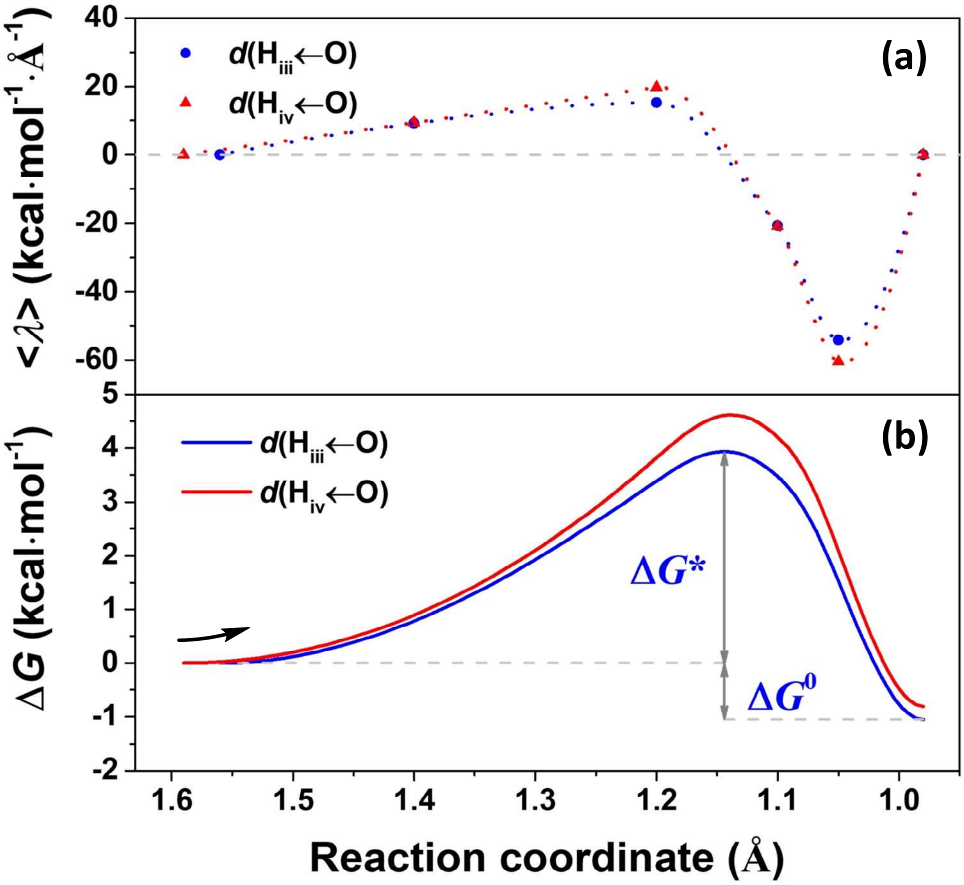

Figure S2. (a) Time-averaged constraint force represented by the Lagrangian multiplier <λ> computed for each constrained MD simulation as a function of the reaction coordinate d(H_{iii}⟵O) and d(H_{iv}⟵O) in the two-channel model. The mean force at the equilibrium distance d(H_{iii}⟵O) = 1.56 Å/d(H_{iv}⟵O) = 1.59 Å and d(H_{iii}⟵O) = 0.98 Å/d(H_{iv}⟵O) = 0.98 Å evaluated in the FMD simulations before and after the reaction, corresponding to the initial and final states along the reaction coordinates d(H_{iii}⟵O) and d(H_{iv}⟵O) respectively, have been assumed to be zero. The Akima splines (100 points) is used to interpolate the mean forces. **(b)** Free energy profile along the reaction coordinates d(H_{iii}⟵O) and d(H_{iv}⟵O) computed from thermodynamic integration.

Table S1. The calculated activation free energy barrier (ΔG^* in kcal mol^{-1}), reaction driving force (ΔG^0 in kcal mol^{-1}), and the reaction rate (k in s^{-1}) corresponding to the reaction coordinates d(H_{iii}⟵O) and d(H_{iv}⟵O).

Reaction coordinate	ΔG^*	ΔG^0	k
d(H_{iii}⟵O)	3.9	-1.1	8.9×10^9
d(H_{iv}⟵O)	4.6	-0.8	2.8×10^9

The obtained free energy profiles for these two PCET processes reported in Figure S5 show similar activation free energy barriers $\Delta G^* \approx 3.9$ kcal mol^{-1} (0.17 eV) and $\Delta G^* \approx 4.6$ kcal mol^{-1} (0.20 eV), corresponding to the reaction coordinates $d(H_{iii}\leftarrow O)$ and $d(H_{iv}\leftarrow O)$ respectively in the two-channel model (see Table S1). This is consistent with the comparable activation barriers of the first and second catalytic PCET steps in the one-channel model[2, 7]. The maximum of the free energy profile corresponds to a reaction coordinate $d(H_{iii}\leftarrow O)/d(H_{iv}\leftarrow O)$ of 1.14/1.14 Å, suggesting an identified transition state with a smaller $H_{iii}\cdots O/H_{iv}\cdots O$ distance compared to that of the first/second catalytic PCET step in one-channel model. This concurrent event of the first two PCET processes is found to be fast and exothermic with the negative driving forces and high reaction rates $\Delta G^0 \approx -1.1$ kcal mol^{-1} (0.05 eV)/$k \approx 8.9\times10^9$ s^{-1} and $\Delta G^0 \approx -0.8$ kcal mol^{-1} (0.03 eV)/$k \approx 2.8\times10^9$ s^{-1} corresponding to the reaction coordinates $d(H_{iii}\leftarrow O)$ and $d(H_{iv}\leftarrow O)$, respectively, indicating that the selected NDI dyes are able to cooperatively drive this concurrent event in such a dye−WOC−dye system.

S3. COMPUTATIONAL DETAILS

S3.1 Geometry Optimization at DFT Level

The OPBE exchange-correlation functional[1] and the TZP (triple-ζ polarized) Slater-type basis set[2] were employed in the geometry optimization of the initial state of the dye−WOC−dye complex. The OPBE functional has shown to be accurate in describing transition-metal complexes, including Ru-based WOCs.[8] In the geometry optimization, the continuous solvation model (COSMO[4]) for water was used. These calculations are performed with the ADF software package.[3]

S3.2 Simulation Box

To obtain a realistic description of the catalytic reaction step, the solvent was explicitly introduced in the simulations. The solvent environment for the CPMD simulations was generated using Discovery Studio 2.5.[9] The solvent was equilibrated for 0.2 ns using the TIP3P model implemented in the CHARMM force field and CFF partial charge parameters at 300 K,[10] while the dye−$[WOC]^{2+}$−dye complex was kept fixed. The volume was then adjusted using constant pressure for 0.2 ns, after which the system was further allowed to evolve with constant volume for 2 ns. Periodic boundary conditions are applied with a time step of $\delta t = 5$ a.u. (1 a.u. = 0.0242 fs).

S3.3 Free Energy Profile

To estimate the activation free energy barrier of the catalytic reaction step involving the O–O bond formation that is unlikely to occur spontaneously during the typical AIMD simulation time scale, constrained MD and the so-called Blue Moon approach were employed as a rare event simulation technique.[6] The reaction coordinate (in this case the distance between two oxygen atoms O_i and O_{ii}, $d(O_i \leftarrow O_{ii})$, as shown in Scheme 1) is constrained to a series of fixed values x in range of 2.5 − 1.6 Å after the initial equilibrium simulation and subsequent photooxidation of two NDI dyes along this reaction pathway. A time-averaged constraint force $\langle\lambda\rangle_x$ for each value of the reaction coordinate x is obtained, which should be equal to zero at an equilibrium or transition state. Based also on our previous work on a similar supramolecular complex, we can safely assume that for a value of the reaction coordinate $d(O_i \leftarrow O_{ii}) = 3.0$ Å the system is in an equilibrium state with $\langle\lambda\rangle_{3.0\,Å}$ equals to zero.[7] The activation free energy barrier for this catalytic step is then established by interpolating the mean forces with a 100-

point Akima splines function and integrating the signed forces <λ>$_x$ along the reaction path.[11] Trajectory analysis and visualization for the CPMD output were carried out using VMD.[12]

S3.4 Reaction Rate

The computed activation free energy barrier can be used to evaluate to what extent the geometry modification accelerates the rate of the third water oxidation step involving the O−O bond formation. According to transition state theory[13], the reaction rate (k) determined by the activation energy barrier (ΔG^*) can be expressed as

$$k = \frac{k_B T}{h} \cdot e^{-\frac{\Delta G^*}{RT}}, \tag{1}$$

Where ΔG^* represents the activation free energy barrier, k_B, h, R and T are the Boltzmann constant, the Planck constant, the universal gas constant and thermodynamic temperature, respectively. One should keep in mind that in the DFT-based MD simulations protons are treated classically and thus proton tunneling effects are neglected. In the current calculation, only the activation energy barrier is considered as a main factor governing the reaction rate.

S4. Molecular orbital and electronic structure of ^{3}(NDI1$^{+\bullet}$–[Ru^{IV}=O]$^{2+}$–NDI2$^{+\bullet}$)

Table S2. Selected frontier molecular orbital energy levels and energy difference between the highest SOMO WOC and SOMO dye1/dye2 (ΔE_{SOMO-1}/ΔE_{SOMO-2}, in eV) of intermediate ^{3}(NDI1$^{+\bullet}$–[Ru^{IV}=O]$^{2+}$–NDI2$^{+\bullet}$) after the photooxidation of two NDI dyes.[a]

**Intermediate**		**^{3}(NDI1$^{+\bullet}$–[Ru^{IV}=O]$^{2+}$–NDI2$^{+\bullet}$)**
		↓ ↑ ↑ ↑
**Energy level**	**Orbital**	**Energy**
**HOMO** (*SOMO WOC*)	α	−6.302
**HOMO-1** (*SOMO dye1*)	β	−6.482
**HOMO-2** (*SOMO dye2*)	α	−6.509
**HOMO-3** (*dye*)	α	−6.773
**HOMO-4** (*dye*)	β	−6.806
**HOMO-5** (*SOMO-1 WOC*)	α	−6.838
**ΔE_{SOMO-1}**		0.180
**ΔE_{SOMO-2}**		0.207

[a]Only the unpaired electrons are indicated by vertical arrows explicitly (green for unpaired electron localized on the catalyst and blue for unpaired electron on the oxidized NDI$^{+\bullet}$). SOMO represents the singly occupied molecular orbital.

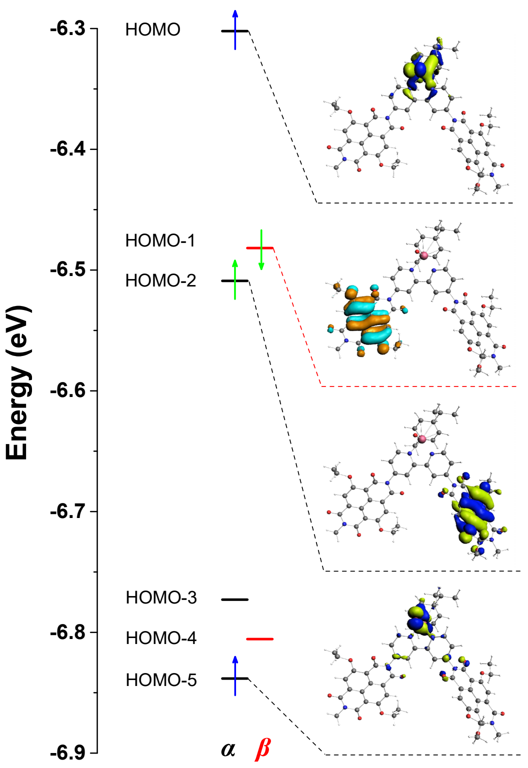

Figure S3. Selected frontier molecular orbitals of intermediate ^{3}(NDI1$^{+\bullet}$–[Ru^{IV}=O]$^{2+}$–NDI2$^{+\bullet}$) after the photooxidation of two NDI dyes computed with the ADF program using the OPBE functional and the TZP basis set. The left (black) and right (red) orbital energy levels refer to the α orbitals and β orbitals, respectively. Only the unpaired electrons are indicated by vertical arrows explicitly (blue for unpaired electron localized on the catalyst and green for the unpaired electron on the oxidized NDI$^{+\bullet}$). See **Table S2** for the molecular energy levels and the energy difference between SOMO WOC and SOMO dye.

S5. DFT calculation of ^{5}(NDI1$^{+\bullet}$–[Ru^{IV}=O]$^{2+}$–NDI2$^{+\bullet}$)

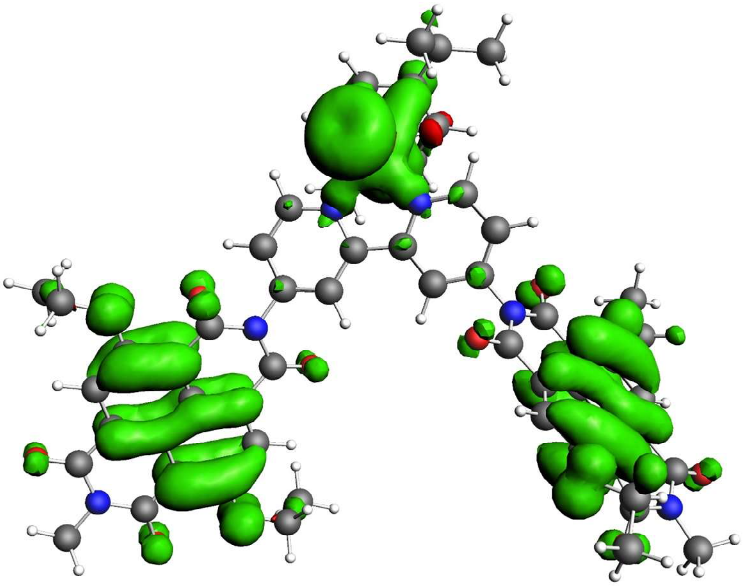

Figure S4. The spin density isosurface (green) of ^{5}(NDI1$^{+\bullet}$–[Ru^{IV}=O]$^{2+}$–NDI2$^{+\bullet}$) in the quintet state after the photooxidation of the two NDI dyes computed with the ADF program using the OPBE functional and the TZP basis set. The triplet and the quintet state of NDI1$^{+\bullet}$–[Ru^{IV}=O]$^{2+}$–NDI2$^{+\bullet}$ have the same bond energy (see **Table S3**).

Table S3. Bond energy E_{bond} (in kcal mol^{-1}) of the triplet state ^{3}(NDI1$^{+\bullet}$–[Ru^{IV}=O]$^{2+}$–NDI2$^{+\bullet}$) and quintet state ^{5}(NDI1$^{+\bullet}$–[Ru^{IV}=O]$^{2+}$–NDI2$^{+\bullet}$) of NDI1$^{+\bullet}$–[Ru^{IV}=O]$^{2+}$–NDI2$^{+\bullet}$ after the photooxidation of the two NDI dyes.[a]

**Intermediate**	***E*bond**
**^{3}(NDI1$^{+\bullet}$–[Ru^{IV}=O]$^{2+}$–NDI2$^{+\bullet}$)**<br>⇅ ↑ ↑ ↑	-19668.81
**^{5}(NDI1$^{+\bullet}$–[Ru^{IV}=O]$^{2+}$–NDI2$^{+\bullet}$)**<br>↑ ↑ ↑ ↑	-19668.81

[a]Only the unpaired electrons are indicated by vertical arrows explicitly (green for unpaired electron localized on the catalyst and blue for unpaired electron on the oxidized NDI$^{+\bullet}$).

S6. Spin density after photooxidation of two NDI dyes

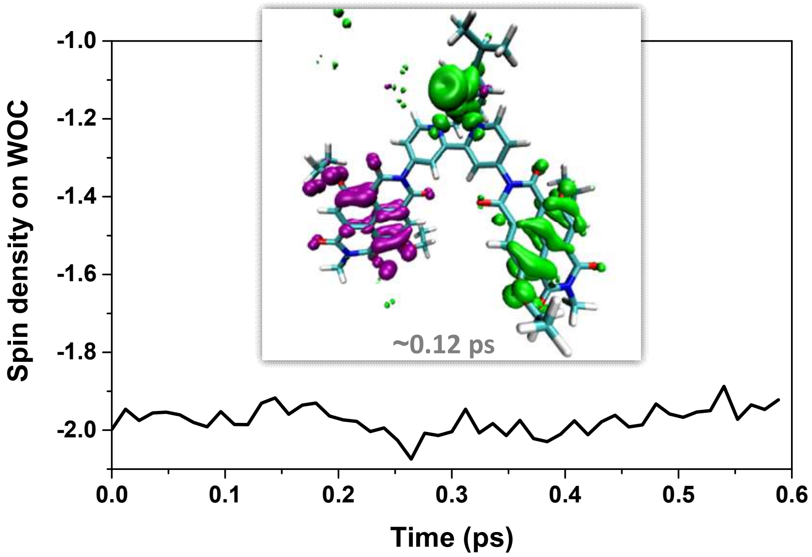

Figure S5. Spin density integrated over the part of the simulation box including the WOC along the free MD trajectory after the photooxidation of two NDI dyes. Inset shows the spin density isosurface computed at a snapshot taken at ~0.12 ps, clearly indicating two unpaired α electrons (↑ in green) localized on the catalyst, one unpaired β electron on NDI1 (↓ in purple), and one unpaired α electron on NDI2 (↑ in green). See **Scheme 1** for the atomic labelling. The water molecules are omitted for clarity and only the intermediate $^3(NDI1^{+\bullet}–[Ru^{IV}=O]^{2+}–NDI2^{+\bullet})$ is shown explicitly.

S7. Proton transfer (PT) step during the constrained 1.8 Å simulation

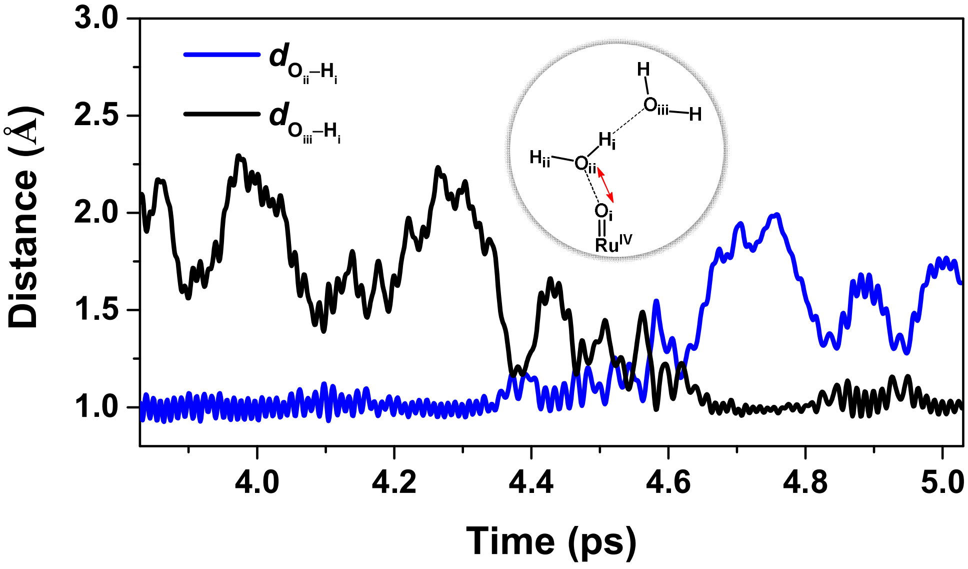

Figure S6. Time evolution of the geometrical parameters $d_{O_{ii}-H_i}$ (blue line) and $d_{O_{iii}-H_i}$ (black line) along the constrained MD trajectory with $d(O_i \leftarrow O_{ii})$ = 1.8 Å. The inset shows the schematic structure of the first two water molecules along the hydrogen-bonding network coordinated to the oxygen ligand. The time range is consistent with that in **Figure 1**.

S8. Time-averaged constraint forces

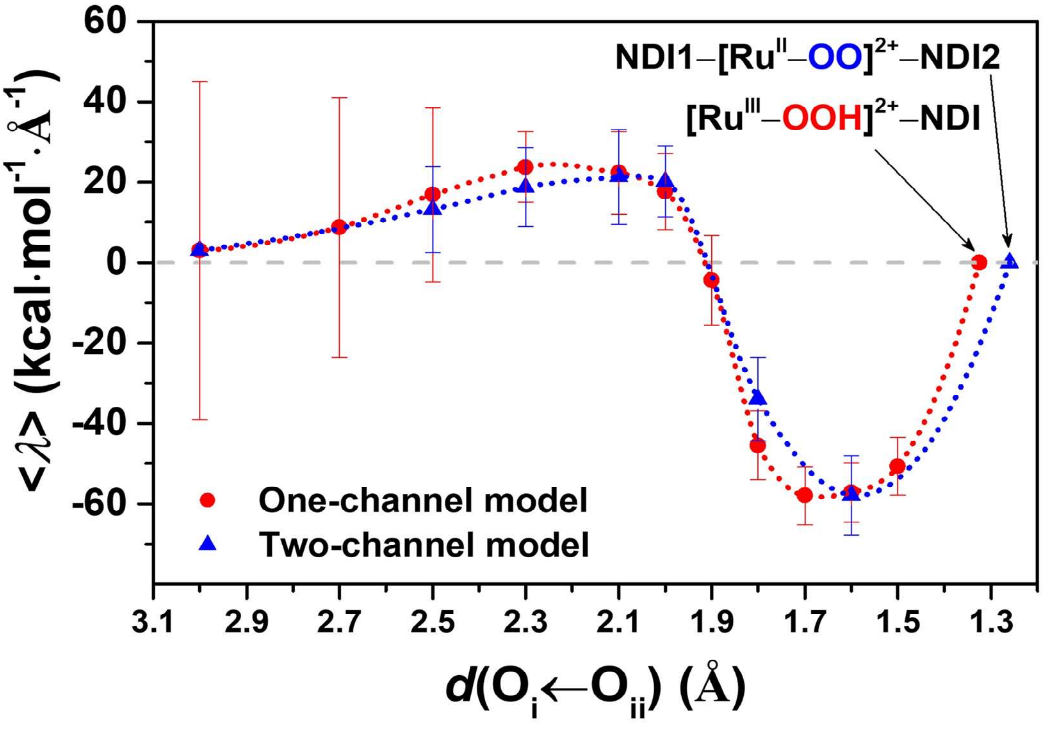

Figure S7. Time-averaged constraint force represented by the Lagrangian multiplier <λ> computed for each constrained MD simulation as a function of the reaction coordinate $d(O_i \leftarrow O_{ii})$ in the two-channel model. The Akima splines (100 points) is used to interpolate the mean forces. The final intermediates corresponding to the MD simulations for the one-channel and two-channel models are both indicated. The time-averaged constraint forces obtained in the one-channel model from a previous study is also presented for comparison (see Ref. [7]). The error bars indicate the standard deviations.

References

[1] M. Swart, A. W. Ehlers, K. Lammertsma, *Mol. Phys.* **2004**, *102*, 2467-2474.

[2] A. Monti, J. M. de Ruiter, H. J. M. de Groot, F. Buda, *J. Phys. Chem. C* **2016**, *120*, 23074-23082.

[3] a) G. te Velde, F. M. Bickelhaupt, E. J. Baerends, C. Fonseca Guerra, S. J. A. van Gisbergen, J. G. Snijders, T. Ziegler, *J. Comput. Chem.* **2001**, *22*, 931-967; b) ADF2017, SCM, Theoretical Chemistry, Vrije Universiteit, Amsterdam, The Netherlands, http://www.scm.com.

[4] a) A. Klamt, *J. Phys. Chem.* **1995**, *99*, 2224-2235; b) A. Klamt, V. Jonas, *J. Chem. Phys.* **1996**, *105*, 9972-9981.

[5] CPMD, http://www.cpmd.org, Copyright IBM Corp., 1990−2019; Copyright MPI für Festkörperforschung Stuttgart, 1997−2001.

[6] a) B. Ensing, E. J. Meijer, P. E. Blöchl, E. J. Baerends, *J. Phys. Chem. A* **2001**, *105*, 3300-3310; b) G. Ciccotti, M. Ferrario, *Mol. Simul.* **2004**, *30*, 787-793; c) F. Costanzo, R. G. Della Valle, *J. Phys. Chem. B* **2008**, *112*, 12783-12789.

[7] Y. Shao, J. M. de Ruiter, H. J. M. de Groot, F. Buda, *J. Phys. Chem. C* **2019**, *123*, 21403-21414.

[8] a) A. T. P. Carvalho, M. Swart, *J. Chem. Inf. Model.* **2014**, *54*, 613-620; b) A. R. Groenhof, A. W. Ehlers, K. Lammertsma, *J. Am. Chem. Soc.* **2007**, *129*, 6204-6209; c) J. Conradie, A. Ghosh, *J. Chem. Theory and Comput.* **2007**, *3*, 689-702; d) J. L. Vallés-Pardo, M. C. Guijt, M. Iannuzzi, K. S. Joya, H. J. M. de Groot, F. Buda, *ChemPhysChem* **2012**, *13*, 140-146.

[9] Accelrys Software Inc. *Discovery Studio Modeling Environment*, Accelrys Software Inc.: San Diego, 2012.

[10] B. R. Brooks, R. E. Bruccoleri, B. D. Olafson, D. J. States, S. Swaminathan, M. Karplus, *J. Comput. Chem.* **1983**, *4*, 187-217.

[11] a) W. K. d. Otter, W. J. Briels, *J. Chem. Phys.* **1998**, *109*, 4139-4146; b) M. Sprik, G. Ciccotti, *J. Chem. Phys.* **1998**, *109*, 7737-7744; c) L. Bernasconi, E. J. Baerends, M. Sprik, *J. Phys. Chem. B* **2006**, *110*, 11444-11453; d) L. Bernasconi, A. Kazaryan, P. Belanzoni, E. J. Baerends, *ACS Catal.* **2017**, *7*, 4018-4025.

[12] a) W. Humphrey, A. Dalke, K. Schulten, *J. Mol. Graphics* **1996**, *14*, 33-38; b) VMD - Visual Molecular Dynamics. *Theoretical Chemistry and Computational Biophysics Group*, University Of Illinois: Urbana, 2016.

[13] a) H. Eyring, *J. Chem. Phys.* **1935**, *3*, 107-115; b) K. J. Laidler, M. C. King, *J. Phys. Chem.* **1983**, *87*, 2657-2664; c) E. Pollak, P. Talkner, *Chaos* **2005**, *15*, 026116.
